# Reliability and safety of minimally invasive esophagectomy after neoadjuvant chemoradiation: a retrospective study

**DOI:** 10.1186/s13019-019-0920-0

**Published:** 2019-05-28

**Authors:** Guangyuan Liu, Yongtao Han, Lin Peng, Kangning Wang, Yu Fan

**Affiliations:** 1 Department of Thoracic Surgery, Sichuan Cancer Hospital and Institute, No.55, Section4, South Renmin Road, Chengdu, 610041 China; 2 Department of Radiation Oncology, Sichuan Cancer Hospital and Institute, No.55, Section4, South Renmi Road, Chengdu, 610041 China

**Keywords:** Locally advanced esophageal cancer, Neoadjuvant chemoradiotherapy (NCRT), Minimally invasive esophagectomy (MIE), Esophageal squamous cell carcinoma (ESCC)

## Abstract

**Background:**

Thoracic surgeons have recognized the advantages of minimally invasive esophagectomy (MIE). However, MIE for locally advanced esophageal cancer after neoadjuvant chemoradiotherapy (NCRT) is controversial. This study aimed to nvestigate and summarise the reliability and safety of MIE after NCRT.

**Methods:**

We retrospectively analyzed the perioperative outcomes of patients with locally advanced esophageal cancer who underwent minimally invasive esophagectomy after neoadjuvant chemoradiotherapy from January 2016 to January 2018, and compared them with patients who underwent MIE alone during the same period.

**Results:**

In total, 107 patients were eligible for the study. Forty-four patients underwent MIE after NCRT (CRM), and 63 patients underwent MIE alone (MA). The surgical duration (253.59 ± 47.51 vs. 222.86 ± 42.86 min), intraoperative blood loss (164.55 ± 109.09 vs. 146.19 ± 112.89 ml), number of lymph nodes resected (18.36 ± 8.01 vs. 22.10 ± 12.03), duration of the postoperative hospital stay (12.84 ± 6.57 vs. 14.60 ± 8.48 days), postoperative intubation time (5.68 ± 3.08 vs. 6.54 ± 4.97 days), total incidence of complications (34.10% vs. 31.7%), and R0 resection rate (95.45% vs. 96.83%) had no significant difference. The incidence of arrhythmia was higher in CRM (*P* < 0.02). No mortality occurred postoperatively within 30 days in either group.

**Conclusion:**

Minimally invasive esophagectomy after neoadjuvant chemoradiotherapy is a feasible, safe, and beneficial for postoperative recovery of patients.

**Electronic supplementary material:**

The online version of this article (10.1186/s13019-019-0920-0) contains supplementary material, which is available to authorized users.

## Background

Esophageal cancer is one of the most common malignant tumours in the world, with the squamous cell carcinoma type accounting for 90% of the cases [1, 2]. Comprehensive treatment is recommended for locally advanced esophageal cancer. A meta-analysis by Gebski et al. [3] included multiple randomized comparison studies with a total of 1724 patients and showed that neoadjuvant chemoradiotherapy (NCRT) increased the 2-year survival rate by 7%. Van Hagen et al. reported that the 5-year survival rate for patients receiving NCRT followed by surgery was higher than that of patients undergoing surgery alone (47% vs 34%, respectively) [4]. The results of the chemoradiotherapy for esophageal cancer followed by surgery [5] showed that NCRT increased pathologically complete response (pCR) rates and improved the prognosis for patients noticeably. Preoperative chemoradiotherapy increased the radical resection rate, progression-free survival and overall survival. The study is of great significance to the treatment of esophageal cancer in China.

The safety and feasibility of minimally invasive esophagectomy (MIE) are well-recognized, and the long-term survival rate is similar to open surgery. MIE is significantly better than open surgery for reducing postoperative complications, enhancing postoperative recovery, and improving the postoperative quality of life [6]. Biere et al. reported that minimally invasive surgery resulted in less blood loss, a lower rate of pulmonary infection (9% vs. 29%), and a shortened hospital stay than open surgery, but it required a longer operative duration than open surgery.These conclusions have also been confirmed in other meta-analyses [7–11].

Some scholars consider that preoperative chemoradiotherapy leads to local tissue oedema, fibrosis, unclear anatomical stratification, and decreased immunity, which increases the difficulty of minimally invasive surgery and perioperative complications. Therefore, could MIE have superior outcomes on patients who received induction radiation and chemotherapy compared to those who only had MIE?There are numerous studies that compare MIE with open esophagectomy, with or without NCRT. Fewer studies have compared MIE after NCRT with MIE alone. Is it reliable and safe for patients to have MIE after a neoadjuvant chemoradiation?In this retrospective study, we analysed patients who underwent MIE after NCRT due to locally advanced esophageal cancer from January 2016 to January 2018. Then, we compared the data of the patients with those who underwent MIE only; we found it achieved good short-term results.

## Methods

### Patients

**Fig. 1:**
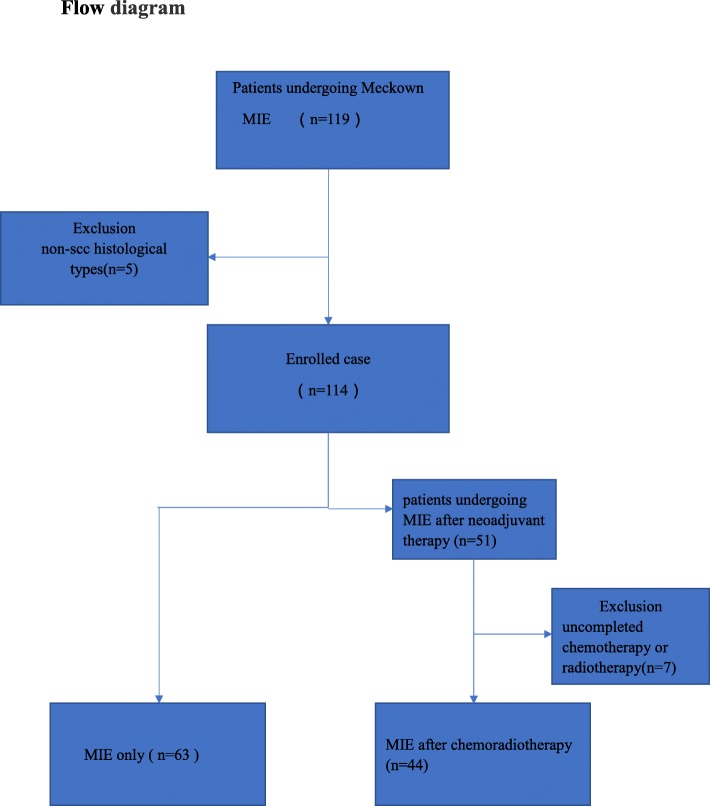
Flow diagram

A total of 107 patients were eligible for this analysis. The flow chart is shown in Fig. 1. Between January 2016 and January 2018, all the patients were diagnosed with esophageal squamous cell carcinoma, and assessed with cervical and endoscopic ultrasonography, thoracic and abdominal enhanced computed tomography, or positron emission tomography (PET). The clinical stages were stages II- IV (T2-4 N0-2 M0). Forty-four patients underwent a McKeown MIE after chemoradiotherapy, and 63 patients underwent a McKeown MIE only. All the patients signed informed consent before any treatment (Fig. 1).

### Neoadjuvant chemoradiotherapy

Preoperative radiotherapy was administered using the Intensity Modulated Radiation Therapy (IMRT) technique, and each patient received conventional fractionation of 200 cGy/d 5 days per week for 4 weeks, resulting in a total dose of 40 Gy. The tumour target area included the primary esophageal tumour and metastatic lymph nodes, and the clinical target area included subclinical lesions (3 cm of normal oesophagus above and below the oesophageal tumour and the corresponding para-esophageal lymphatic drainage area).

Preoperative chemotherapy included a regimen of paclitaxel plus cisplatin. Each patient received paclitaxel at a dose of 135 mg/m^2^ intravenously on day 1 and cisplatin at a dose of 25 mg/m^2^ intravenously on day 1–3. The chemotherapy lasted for 2 cycles.

### Surgical procedure

Four to six weeks after completion of the neoadjuvant chemoradiotherapy, the patients were examined using gastrointestinal endoscopy, endoscopic ultrasonography, cervical ultrasonography, thoracic, and abdominal enhanced CT or PET. All the patients were reassessed according to the Response Evaluation Criteria in Solid Tumours (RECIST). The clinical efficacy was evaluated as follows: 19 cases with a partial response (PR), 21 cases with a complete response (CR), and 4 cases with stable disease (SD). No further surgical contraindications were observed during the reassessment, and the McKeown MIE were performed (Additional file 1: Surgery video clip).

The parameters evaluated during surgery were: surgical duration, intraoperative blood loss, the number of lymph nodes resected, surgical resection status, postoperative indwelling duration of the chest tube, duration of the postoperative hospital stay, and the incidence of perioperative complications including arrhythmia, pulmonary infection, recurrent laryngeal nerve injury, anastomotic fistula, and mortality. Clinic staging, postoperative pathological staging, and tumour location followed the Union for International Cancer Control (UICC) esophageal cancer staging manuals, 8th edition.

### Statistical analysis

The data were analysed using the SPSS 23.0 statistical software. Comparisons between the groups were performed with the χ^2^ test and Fisher’s exact test for categorical parameters, while with Student’s t test or analysis of variance test for continuous variables. A *P* value of less than 0.05 was considered to be significant.

## Results

Among all the patients, 44 were in the NGRT+MIE group and 63 were in the MIE alone group. Demographic and clinical data were compared (Table 1).Of the 44 patients in the NCRT+MIE group, 38 were men, 6 were women, and the average age was 59.45 years**.**Tumours were localized in the upper section on 8 cases, the middle section on 33 cases, and the lower section on 3 cases. The clinical stages were stages II-IV. The surgical duration time was 253.59 ± 47.51 min, intraoperative blood loss was 164.55 ± 109.09 mL, and the number of resected lymph nodes was 18.36 ± 8.01. The postoperative intubation time was 5.68 ± 3.08 days, and the duration of the postoperative hospital stay was 12.84 ± 6.57 days. Five patients were converted to open thoracotomy during the operation. The completion rate of MIE was 88.64%. Among the patients, 42 cases achieved R0 resection, 1 case achieved R1 resection, and 1 case achieved R2 resection, with an R0 resection rate of 95.45%. The overall incidence of complications was 34.10%, including 2 cases of hoarseness, 3 cases of anastomotic fistula, 8 cases of pulmonary infection, 1 case of chylothorax, and 8 cases of arrhythmia. No incision infections or thrombosis-related events were noted, and no perioperative mortality occurred. The postoperative pathologic stages (ypTNM staging) were stages I-IVA, with a complete pathological response (pCR) rate of 29.55%.

**Table 1:** Patient demographic, Clinical, and preoperative data

	NCRT + MIE	MIE alone
Age(mean)	47–70 (59.45)	44–73 (60.06)
Male/Female	38/6	47/16
Smoking history		
yes	35 (79.5%)	44 (69.8%)
no	9 (20.5%)	19 (30.2%)
Drinking history		
yes	34 (77.3%)	42 (66.7%)
no	10 (22.7%)	21 (33.3%)
COPD		
yes	5 (11.4%)	8 (12.7%)
no	39 (88.6%)	55 (87.3%)
Location of primary tumor		
Upper thoracic	8 (18.2%)	10 (15.9%)
Middle thoracic	33 (75%)	37 (58.7%)
Lower thoracic	3 (6.8%)	16 (25.4%)
T-classification		
T1	0	8 (12.7%)
T2	1 (2.3%)	34 (54.0%)
T3	29 (65.9%)	21 (33.3%)
T4	14 (31.8%)	0
N-classification		
N0	1 (2.3%)	36 (57.1%)
N+	43 (97.7%)	27 (42.9%)

Of the 63 patients in the MIE alone group, 47 were men and 16 were women, and the average age was 60.06 years. Tumours were localized in the upper section on 10 cases, the middle section on 37 cases, and the lower section on16 cases. The clinical stages were stages I-III. The surgical duration time was 222.86 ± 42.86 min, intraoperative blood loss was 146.19 ± 112.89 ml, and the number of resected lymph nodes was 22.10 ± 12.03. The postoperative intubation time was 6.54 ± 4.97 days, and the duration of the postoperative hospital stay was 14.60 ± 8.48 days. Five patients were converted to open thoracotomy during surgery. The completion rate of MIE was 92.06%. Among the patients, 61 cases achieved R0 resection, 2 cases achieved R1 resection, and no cases achieved R2 resection, R0 resection rate was 95.45%. The overall incidence of complications was 31.75%, including 6 cases of recurrent laryngeal nerve injury, with 3 cases exhibiting hoarseness, 4 cases of anastomotic fistula, 10 cases of pulmonary infection, and 1 case of arrhythmia. No incision infections or thrombosis-related events were noted, and no perioperative mortality occurred. Clinical stage, Pathological stage and response were compared (Table 2).

**Table 2:** Clinical stage, Pathological stage and response

	NCRT+MIE(*n* = 44)	MIE alone(*n* = 63)
Clinical stage		
	II 2 (4%)	I 8 (13%)
	III 25 (57%)	II 51 (81%)
	IV 17 (39%)	III 4 (6%)
Pathological stage	ypTNM	pTNM
	I 21 (47%)	IA 5 (8%)
	II 5 (11%)	IB 2 (3%)
	IIIA 5 (11%)	IIA 11 (18%)
	IIIB 10 (22%)	IIB 19 (31%)
	IVA 4 (9%)	IIIA 17 (26%)
		IIIB 7 (11%)
		IVA 2 (3%)
Response	PR 19 (43%)	
	CR 21 (48%)	
	SD 4 (9%)	
	pCR 13 (29.55%)	

Comparing both groups, surgical duration, intraoperative blood loss, number of lymph nodes, duration of the postoperative hospital stays, postoperative intubation time, total incidence of complications, and R0 resection rate have no significant difference. Incidence of arrhythmia was higher in CRM (*P* < 0.02) (Tables 3 and 4).

**Table 3:** Perioperative outcomes in NCRT + MIE and MIE alone patients

	NCRT+MIE	MIE alone	*P*
Surgical duration (min)	253.59 ± 47.51	222.86 ± 42.86	0.675
Blood loss (mL)	164.55 ± 109.09	146.19 ± 112.89	0.545
Incidence of complications	34.10%	31.75%	0.799
Duration of postoperative hospital stay (d)	12.84 ± 6.57	14.60 ± 8.48	0.721
Duration of intubation (d)	5.68 ± 3.08	6.54 ± 4.97	0.311
Surgery completion rate	88.64%	92.06%	0.549
Numberof lymph nodes resected	18.36 ± 8.01	22.10 ± 12.03	0.075
R0 resection rate	95.45%	96.83%	> 0.99

**Table 4:** Postperative complications of NRT + MIE and MIE alone patients

	NCRT+MIE	MIE alone	*p*
Recurrent laryngeal nerve injury	2 (4.5%)	6 (9.5%)	0.55
Arrhythmia	8 (18.1%)	2 (3.2%)	0.02
Pulmonary infection	8 (18.1%)	10 (15.9%)	0.81
Chylothorax	1 (2.3%)	0	0.41
Anastomotic fistula	3 (6.8%)	4 (6.3%)	> 0.99
Total complications	15 (34.1%)	20 (31.7%)	0.799

## Discussion

The recommended treatment for oesophageal cancer is multidisciplinary therapy, including surgery, radiotherapy, chemotherapy, and targeted therapy. Preoperative chemoradiotherapy is imperative in patients with locally advanced esophageal cancer. Multiple studies around the world have shown that although NCRT combined with surgical treatment for esophageal carcinoma did not reduce the local recurrence rate and distant metastasis rate, such combined treatment can improve the surgical R0 resection rate and long-term survival rate of the patients to some extent when compared with surgery alone [2]. A meta-analysis compared the postoperative survival rate after NCRT combined with surgery and the survival rate with surgery alone and found that while the 1-year survival rate was not significantly different between the two groups, the NCRT group had a higher 2–5-year survival rate, and the 5-year survival rate with chemoradiotherapy was significantly better than that with surgery alone. The 5-year survival rate with sequential chemoradiotherapy did not show any obvious advantages [12]. Another meta-analysis suggests that preoperative chemoradiotherapy and preoperative chemotherapy are significantly better than surgery alone, but the advantages of neoadjuvant chemoradiotherapy and neoadjuvant chemotherapy remain controversial [13]. Endoscopy causes minimal trauma to the patient, which is beneficial for postoperative recovery and reduces the incidence of perioperative complications [14, 15]. Currently, approximately 30% of esophageal cancers are treated with MIE worldwide [16, 17]. However, the selection of a specific surgical procedure in medical centre depends on the technical capacity of the centre, individual differences in patients, and tumour-related characteristics [18]. MIE is currently the preferred technique. However, the application of MIE in patients with esophageal cancer after neoadjuvant therapy remains controversial. MIE has been reported to be feasible for patients with advanced esophageal cancer after neoadjuvant therapy, but the majority of evidence is based on tumours of the lower esophageal segment, most of which are adenocarcinomas. Squamous cell carcinoma has different tumour characteristics, and tumours of the upper and middle esophageal segments are more challenging surgically [19–21]. Compared to open surgery, minimally invasive surgery has the advantages of minimal trauma, a shorter surgical duration, less blood loss, and lower pulmonary infection rates. However, preoperative chemoradiotherapy reduces patients’ immunity, causes local tissue oedema, adhesions, and fibrosis, and it induces anatomical and tissue organization changes [22]. Additionally, advanced esophageal cancer is typically located near the aorta, trachea, and bronchus, and injury to any of these structures will cause serious complications. The retrospective cohort study by Merrit et al. reported that neoadjuvant therapy did not increase postoperative morbidity and mortality, and postoperative complications were often associated with preoperative complications and surgical techniques [23]. A study by Bosch et al. suggested that neoadjuvant therapy increases cardiovascular and pulmonary complications, mainly pneumonia and arrhythmia [24]. A meta-analysis by Waresijiang et al. also showed that perioperative complications and mortality were significantly lower with MIE versus open esophagectomy [ [25]]. Rizk et al. reported that postoperative complications were associated with surgical techniques and that minimally invasive surgery performed by skilled surgeons can significantly reduce surgery-related complications [26].

Based on our experience, the McKeown MIE is feasible, and its safety and reliability are ensured by surgical team collaboration. Advancements in chemoradiotherapy technology have enabled considerable minimization of damage to normal tissues and reduced the associated complications. However, after radiotherapy, the primary tumour site of esophageal cancer and the areas receiving radiotherapy often exhibit necrosis, fibrosis, and organizational alterations, further, the normal tissue gap disappears and transforms into a hard, fibrous plate-like structure. The boundaries of the mediastinum are blurred, with an unclear anatomical level. Adhesive fibrosis is more serious in patients with obvious tumour invasion before radiotherapy. Therefore, such surgical procedures have high technical requirements for surgeons, especially for tumours in the middle and upper thoracic esophagus where resection is more difficult and requires careful planning and refinement. The operation should involve precise isolation with minimal dragging and pushing forces that may cause aortic and tracheal damage. Isolation should start from areas with no lesion and gradually extend to areas with the lesion. The dense adhesions of important organs should be addressed at the end of the procedure to reduce the possibility or extent of damage. An experienced surgeon can use a surgical instrument to push against the tumour tissue to assess the mobility of the lesion and its relationship with the trachea and the aorta. If the lesion invades the aorta, the lesion can be resected together with the aortic adventitia. Extended intubation time may damage the throat and trachea. Balloon dilatation of the left main bronchus affects the exposure of the left recurrent laryngeal nerve lymph node area. Dragging force on the trachea may lead to displacement of the endotracheal tube and result in poor lung collapse, which increases the difficulty of the operation during resection of subcarinal and left recurrent laryngeal nerve lymph nodes [7]. Single-lumen endotracheal intubation results in a better collapse of the tracheal membrane, and the use of an artificial pneumothorax in thoracic surgery further accelerates complete lung collapse. Subsequently, the interstitial adipose tissue gap within the mediastinum and the tissue gap around the lymph nodes are widened to fully expose the surgical field, facilitate isolation, reduce blood loss, decrease the rate of recurrent laryngeal nerve injury, and promote thorough resection of the lymph nodes [8, 27].

Common reasons for conversion to open surgery include total thoracic dense adhesions, technical difficulties since an early stage, and a requirement for dual-lung ventilation to ensure stable intraoperative respiration. In this study, no significant differences were identified in the surgical duration, intraoperative blood loss, the number of lymph node resected, duration of the postoperative hospital stay, postoperative intubation time, and the total complication rate between the two groups (*p* > 0.05). In a study on minimally invasive surgery in patients after NCRT by Mu et al., patients at stage III accounted for 25.1% of the cases, average surgical duration was 330 min, blood loss was 100 mL (100–200 mL), average number of resected lymph nodes was 22, the duration of hospitalization was 16 days, total incidence of complications was 43.4% (33/76), incidence of complications related to surgery was 25%, and the incidence of pulmonary complications was 2.9% [28]. Bagheri et al. carried out a study on pulmonary complications and found that the difficulty of ventilator separation was related to the number of sputum bacteria, while no correlation was found between neoadjuvant therapy and pulmonary complications [29]. In our study of the NCRT+MIE group, 8 cases of arrhythmia were noted, which is significantly more than the 2 cases identified in the surgery alone group (*p* = 0.02). The 8 cases of arrhythmia were paroxysmal supraventricular tachycardia, and they were all converted to sinus rhythm after treatment with no adverse cardiac events. These events may be related to myocardial damage caused by chemoradiotherapy. A study by Day et al. found that neoadjuvant chemoradiotherapy tended to increase the incidence of atrial fibrillation complications (73.7% vs 56.6%, *p* = 0.07) [30]. Previous studies concluded that the ratio of total complications for an esophagectomy was 20.5–63.5%, the incidence of minimally invasive surgery was lower than that of open surgery, and the recovery after minimally invasive surgery was faster than that after open surgery [31–33].

This study had some limitations. The sample size of the study was small. Patients enrolled were not randomly assigned, resulting in selection bias. Some of the patients at clinical stage III refused chemoradiotherapy, and surgery was performed alone. Most patients have not been followed up for 3 years. Although we discuss perioperative outcomes, it will be better when we add survival analysis. In addition, the follow-up continued and our department is conducting a clinical trial (CHICTR-INR-17013584). We hope to obtain more effective data to improve the advantages of MIE for patients with oesophageal cancer.

## Conclusion

In a conclusion, based on the current evidence, minimally invasive esophagectomy after neoadjuvant chemoradiotherapy is feasible, safe, and beneficial for postoperative recovery of patients. This surgical technique is worthy of extensive clinical application.

## Additional file

Additional file 1: Surgical video clip. (MPG 64616 kb)

## Abbreviations

CR: Complete response
ESCC: Esophageal squamous cell carcinoma
IMRT: Intensity Modulated Radiation Therapy
MIE: Minimally invasive esophagectomy
NCRT: Neoadjuvant chemoradiotherapy
pCR: pathologically complete response
PR: Partial response
RECIST: Response Evaluation Criteria in Solid Tumours
SD: Stable disease
UICC: Union for International Cancer Control

## References

[1] Arnold M, Soerjomataram I, Ferlay J, Forman D (2015). Global incidence of oesophageal cancer by histological subtype in 2012. Gut.

[2] Lagergren J, Smyth E, Cunningham D, Lagergren P (2017). Oesophageal cancer. Lancet.

[3] Gebskj V, Burmeister B, Smithers BM (2007). Survival benefits from neoadjuvant chemoradiotherapy or chemotherapy in oesophageal carcinoma:a meta—analysis. Lancet 0ncol.

[4] Van Hagen P, Hulshof MC, van Lanschot JJ, Steyerberg EW, van Berge Henegouwen MI, Wijnhoven BP, Richel DJ, Nieuwenhuijzen GA, Hospers GA, Bonenkamp JJ (2012). Preoperative chemoradiotherapy for esophageal or junctional cancer. N Engl J Med.

[5] Shapiro J, van Lanschot JJB, Hulshof MCCM (2015). Neoadjuvant chemoradiotherapy plus surgery versus surgery alone for oesophageal or junctional cancer (CROSS): long-term results of a randomised controlled trial. Lancet Oncol.

[6] Wang H, Shen Y, Feng M (2015). 0utcomes, quality of life, and survival after esophagectomy for squamous cell carcinoma: a propensity score—matched comparison of operative approaches. J Thomc Cardiovasc Surg.

[7] Biere SS, Cuesta MA, van der Peet DL (2009). Minimally invasive versus open esophagectomy for cancer: a systematic review and meta-analysis. Minerva Chir.

[8] Dantoc M, Cox MR, Eslick GD (2012). Evidence to support the use of minimally invasive esophagectomy for esophageal cancer: a meta-analysis. Arch Surg.

[9] Dantoc MM, Cox MR, Eslick GD (2012). Does minimally invasive esophagectomy (MIE) provide for comparable oncologic outcomes to open techniques? A systematic review. J Gastrointest Surg.

[10] Watanabe M, Baba Y, Nagai Y (2013). Minimally invasive esophagectomy for esophageal cancer: an updated review. Surg Today.

[11] Lagarde SM, Vrouenraets BC, Stassen LP (2010). Evidence-based surgical treatment of esophageal cancer: overview of highquality studies. Ann Thorac Surg.

[12] Chen WQ, Zheng RS, Zhang SW, Zeng HM, Zuo TT, Jia MM (2015). Report of cancer incidence and mortality in China, 2012. China Cancer.

[13] Sjoquist KM, Burmeister BH, Smithers BM (2011). Survival after neoadjuvant chemotherapy or chemoradiotherapy for resectable oesophageal carcinoma: an updated meta-analysis. Lancet Oncol.

[14] Little AG, Lerut AE, Harpole DH, Hofstetter WL (2014). The Society of Thoracic Surgeons practice guidelines on the role of multimodality treatment for Cancer of the esophagus and gastroesophageal junction. Ann Thorac Surg.

[15] Biere SS, van Berge Henegouwen MI (2012). Minimally invasive versus open oesophagectomy for patients with oesophageal cancer: a multicentre, open-label, randomised controlled trial. Lancet.

[16] Boone J, Livestro DP, Elias SG (2009). International survey on esophageal cancer: part I surgical techniques. Dis Esophagus.

[17] National Oesophago-Gastric Cancer Audit (2010). An Audit of the care received by people with Oesophago-gastric Cancer in England and Wales.

[18] Javidfar J, Bacchetta M, Yang JA (2012). The use of a tailored surgical technique for minimally invasive esophagectomy. J Thorac Cardiovasc Surg.

[19] Bakhos C, Oyasiji T, Elmadhun N (2014). Feasibility of minimally invasive esophagectomy after neoadjuvant chemoradiation. J Laparoendosc Adv Surg Tech A.

[20] Tapias LF, Mathisen DJ, Wright CD (2016). Outcomes with open and minimally invasive Ivor Lewis Esophagectomy after neoadjuvant therapy. Ann Thorac Surg.

[21] Warner S, Chang YH, Paripati H (2014). Outcomes of minimally invasive esophagectomy in esophageal cancer after neoadjuvant chemoradiotherapy. Ann Thorac Surg.

[22] Su Murphy CC, Correa AM, Ajani JA, Komaki RU (2013). Rgery is an essential component of multimodality therapy for patients with locally advanced esophageal adenocarcinoma. J Gastrointest Surg.

[23] Merritt RE (2011). Morbidity and mortality after esophagectomy following neoadjuvant chemoradiation. Ann Thorac Surg.

[24] Bosch DJ (2014). Impact of neoadjuvant chemoradiotherapy on postoperative course after curative-intent transthoracic esophagectomy in esophageal cancer patients. Ann Surg Oncol.

[25] Yibulayin W, Abulizi S, Lv H, Sun W (2016). Minimally nvasive oesophagectomy versus open esophagectomy for resectable esophageal cancer: a meta-analysis. World J Surg Oncol.

[26] Rizk NP, Bach PB, Schrag D, Bains MS, Turnbull AD, Karpeh M, Brennan MF, Rusch VW (2004). The impact of complications on outcomes after resection for esophageal and gastroesophageal junction carcinoma. J Am Coll Surg.

[27] Taniyama Y, Nakamura T, Mitamura A (2013). A strategy for supraclavicular lymph node dissection using recurrent laryngeal nerve lymph node status in thoracic esophageal squamous cell carcinoma. Ann Thorac Surg.

[28] Mu J-W, Gao S-G, Xue Q, Mao Y-S, Wang D-L, Zhao J, Gao Y-S, Huang J-F, He J (2015). Updated experiences with minimally invasive McKeown esophagectomy for esophageal cancer. World J Gastroenterol.

[29] Bagheri R (2012). The effect of neoadjuvant chemoradiotherapy on airway colonization and postoperative respiratory complications in patients undergoing oesophagectomy for oesophageal cancer. Interact Cardiovasc Thorac Surg.

[30] Day RW, Jaroszewski D, Chang YH, Ross HJ, Paripati H, Ashman JB, Rule WG, Harold KL (2016). Incidence and impact of postoperative atrial fibrillation after minimally invasive esophagectomy. Dis Esophagus.

[31] Biere SS, van Berge HM, Maas KW, Bonavina L, Rosman C, Garcia JR, Gisbertz SS, Klinkenbijl JH, Hollmann MW, de Lange ES (2012). Minimally invasive versus open oesophagectomy for patients with oesophageal cancer: a multicentre, open-label, randomised controlled trial. Lancet.

[32] Nagpal K, Ahmed K, Vats A, Yakoub D, James D, Ashrafian H, Darzi A, Moorthy K, Athanasiou T (2010). Is minimally invasive surgery beneficial in the management of esophageal cancer? A meta-analysis. Surg Endosc.

[33] Verhage RJ, Hazebroek EJ, Boone J, Van Hillegersberg R (2009). Minimally invasive surgery compared to open procedures in esophagectomy for cancer: a systematic review of the literature. Minerva Chir.

